# To Whom National Culture Matters: An Analysis of Mentions of Countries in Titles and Abstracts in Social and Personality Psychology and Related Fields From 1990 to 2020

**DOI:** 10.1002/ijop.70275

**Published:** 2026-10-02

**Authors:** Igor de Almeida, Yukiko Uchida, Kongmeng Liew, Lean Franzl Lim Yao, Eiji Aramaki

**Affiliations:** ^1^ Institute for the Future of Human Society Kyoto University Kyoto Japan; ^2^ Faculty of Science University of Canterbury Christchurch New Zealand; ^3^ Social Computing Laboratory Nara Institute of Science and Technology Ikoma Japan

**Keywords:** culture, diversity in research, social psychology, WEIRD

## Abstract

Culture significantly influences human psychology, making it essential for psychological research, particularly in social psychology, to acknowledge its importance. However, the majority of psychological research ignores or marginalises culture and context. This is problematic because, despite representing a small portion of humanity (specifically Western, Educated, Industrialized, Rich and Democratic (WEIRD) populations) mainstream psychology is published as if it represented the whole humanity. Among the subfields of psychology, social psychology is arguably the one in which this issue should be least prevalent. To investigate this, we conducted a study on titles and abstracts of articles from 69 social psychology journals published between 1990 and 2020, examining how cultural diversity and awareness of its importance changed over time. The results indicate that cultural awareness is more prominent in journals focused on culture, international or local issues; while remaining almost entirely absent in journals focused on theory, reviews and business. Furthermore, cultural awareness displays an imbalance regarding culture: while WEIRD mentions remain most common, mentions of other regions appear to be disproportionately represented or ‘inflated’ in specific contexts. The generalizability of psychological theories will remain impossible if culture is not integrated as a central factor in research.

## 1 Introduction

Throughout the history of psychology, researchers have repeatedly emphasised that culture,^1^ or simply context, is fundamental for understanding the human psyche. Wilhelm Wundt, often praised as the father of experimental psychology, advocated for a psychology that takes into consideration history, context and culture (‘*Völkerpsychologie*’, often translated as ‘folk psychology’). Other prominent psychologists such as Lev Vygotsky, B. F. Skinner and Albert Bandura, despite working with vastly different theories, also emphasised the importance of culture in psychology (Ferrari et al. 2010). Around 1990, researchers in the subfield of cultural psychology proposed concepts that emphasised the importance of culture when conducting psychological experiments and engaging in psychological practice (e.g., self‐construals, cognitive style, tightness and looseness). Those concepts are still influential in psychology nowadays (Heine and Ruby 2010).

Despite these efforts and advancements, most researchers in mainstream psychology have overlooked the importance of culture, conducting most of their studies with samples from Western populations, ignoring other populations and reporting their studies as representative of the whole of humanity.

This issue caught attention in the field, leading researchers to coin the term WEIRD (Western, Educated, Industrialized, Rich and Democratic) to refer to the commonly studied kind of sample in psychology, which is fundamentally different from the majority of the world population, therefore not representative of humanity (Henrich et al. 2010); and WASP (Western Academic Scientific Psychology) to refer to the theoretical canon developed upon those samples (Misra et al. 2025).

There is a growing body of evidence documenting a pervasive lack of cultural diversity in mainstream psychological research, alongside a persistent disregard for its importance. For example, Arnett (2008) reviewed articles from six journals from the *American Psychological Association* from 2003 to 2007, and found that 96% of samples were from Western countries, with 68% coming from the United States alone. In addition, most editors in those APA journals as well as authors who published in them were from Western countries. Thalmayer et al. (2021) showed that little had changed in these journals from 2014 to 2018. Rad et al. (2018) investigated articles published in 2014 and 2017 in *Psychological Science*, a prominent psychology journal with a focus on improving reproducibility and transparency in the field. They found most samples came from Western cultures: 78.4% in 2014 and 70.9% in 2017, with over half from the United States alone. Additionally, 11.4% of the articles from 2014 and 23.4% from 2017 did not report country information of the sample used. Lin and Li (2023) investigated 68 journals in psychology and found that almost half of the authors publishing in those journals, as well as the vast majority of editors and editors‐in‐chief are from the United States. This lack of diversity contributes to a generalisability bias within psychology.

A plausible explanation for why psychology has such lack of diversity is simply because research is predominantly conducted and disseminated within the cultural context of rich Western countries, and it is specifically tailored for those contexts. As argued by Henrich et al. (2010), the objective in these contexts is to construct theoretical frameworks, validate them through experimental design and publish results intended for scientific impact. There is an aspiration towards universalism, but not representativity in research. Research prioritises methodological rigour and theoretical abstraction, overlooking culture and culture (Hornstein and Star 1990). This yields a psychology (WASP), that is framed as a universal science but is, in reality, representative of only a very small portion of humanity, namely the WEIRD (Henrich 2015; Henrich et al. 2010; cf. Sherman 2025). For WASP researchers, cultural detail is often rendered unnecessary when publishing research findings, since it is implied that research comes from only one culture.

Meanwhile, psychological practice and research work differently in other countries. While rich countries may have the financial resources to support substantial portions of psychologists conducting research, most psychologists in developing countries are engaged in applied work, such as health services, rarely working in research (Adair et al. 2002; Zeineddine, Saab, Lášticová, Ayanian, and Kende 2022). Psychological research is published by psychologists from Western countries, then consumed by psychologists inside and outside Western cultures, reinforcing the false idea that Western findings are inherently universal. Paradoxically, findings from other regions are frequently marginalised as culturally specific, which becomes a barrier for Majority World researchers when trying to publish their findings in WASP journals. Beyond scarcity of resources and institutional incentives, Majority World researchers must legitimise the relevance of their work in ways their Western counterparts do not. They often have to prove that their samples are significant and their studies relevant to the field (Zeineddine, Saab, Lášticová, Kende, and Ayanian 2022) and why already established theories need to be reviewed or expanded, failure to do so often results in heightened scepticism towards any theoretical framework that deviates from Western norms (Anjum and Aziz 2024). For example, Jia (2026a) shared his personal experience of being criticised by a senior researcher for highlighting cultural differences in psychological phenomena, illustrating the resistance some scholars face when deviating from mainstream findings. These accounts of WASP resistance to cultural diversity are far from uncommon, but unfortunately often go unreported. This resistance creates an image of WASP journals as unwelcoming to research containing some sort of diversity, especially when the editorial board lacks diversity. For instance, an empirical study found that non‐White researchers feel that journals with only white editorial boards are less likely to accept research from non‐White researchers, and they are less likely to submit their research to those journals (Auelua‐Toomey and Roberts 2022). Acknowledging the importance of culture, which should arguably be the norm (Wang 2016), is often a struggle for those who choose to do so (Zeineddine, Saab, Lášticová, Kende, and Ayanian 2022). Despite these difficulties being somewhat widely known, researchers outside the Western sphere still face them (Raval et al. 2024).

Actions are being taken within the Western sphere to address this issue. A growing number of articles have been published in the last decade, pointing out the problem and showing ways to improve representativeness in research (e.g., Singh et al. 2024; Vaidis et al. 2024; Wang 2016). However, these articles also show that being inclusive is not an easily accomplishable task, and that it is often the case that large international projects serve only the purpose of spreading WASP theories across the world (Jia 2026b).

The lack of cultural inclusiveness and its awareness is evident even in the titles of articles, the very first contact a reader has with a research project. Previous research has shown that samples from WEIRD regions (especially the United States) are less frequently mentioned in titles compared to other regions in psychological research (Cheon et al. 2020; Kahalon et al. 2022), and that researchers from Northern America, Europe, Australia and New Zealand are less likely to mention the country they are affiliated with in titles in social science research (Torres and Alburez‐Gutierrez 2022). These authors attribute this to a cognitive bias among researchers from WEIRD (or Global North) countries, who often overlook culture or consider their cultures as universal or representative of humanity when publishing. In contrast, researchers from other regions (i.e., Global South) must consider their cultures as representative of only one portion of humanity when publishing.

The entire field of psychology seems to be permeated by this issue. However, things may appear slightly better in social psychology,^2^ a subfield in which context and culture are considered essential for understanding humans. As a result, this subfield seems slightly more balanced regarding diversity than other fields within psychology (Arnett 2008; Thalmayer et al. 2021). In addition, review and opinion articles pointing out how social psychology can improve by increasing the diversity of its samples are somewhat frequently published (Henrich et al. 2010; Wang 2016).

The present research aims to elucidate the temporal progress of this subfield over the last decades in terms of awareness of the importance of culture.

### 1.1 Country Mentions in Titles and Abstracts

Researchers are expected to include the most important information regarding their research in their articles' titles and abstracts. This information usually includes the research goal, hypotheses, participant information, methodology, results, conclusions and any other contextual information that the authors deem relevant to the study (American Psychological Association 2017).

A country mention indicates that the authors considered that this information should be displayed and conveyed in the first contact with the reader. There are various reasons why an author may choose to include a country mention in a title or abstract, such as when describing participants of a study (e.g., ‘a large sample of adolescents from Palestine, Germany and Italy’), contextualising a societal issue (e.g., ‘millions of individuals are currently incarcerated in the United States’), providing historical context (e.g., ‘the construction of homosexuality in Buenos Aires, Argentina, 1900–1950’), adapting an instrument or scale to a culture (e.g., ‘the Farsi version of the positive and negative perfectionism scale’) among others.

The absence of a country mention in titles and abstracts does not necessarily mean that the authors believe that country‐level information is irrelevant. It is often the case that this information is just not considered important enough to be in the primary summary of the research. Thus, it is a matter of what constitutes the priority of authors when they publish their research.

In our analyses, we focused on mentions of countries in titles and abstracts as an indicator of the importance of culture in social psychological research. Thus, an article containing a country mention in its title or abstract suggests that culture is highly relevant to its research. Likewise, an article not containing a country mention in its title or abstract suggests that country‐level culture did not reach the level of importance in that research to be included in its opening frame.

Thus, we estimate that the number of country mentions in titles and abstracts reflects the degree of cultural awareness in the articles of a given journal.

### 1.2 The Present Research

The goal of this research project was to analyse the social psychology subfield regarding cultural awareness. This was achieved by investigating: (1) awareness of the importance of culture (cultural awareness) in social psychology journals; (2) diversity in cultural awareness and (3) over time change of cultural awareness and diversity in those journals.

### 1.3 Open Practices

The processed data and analysis code are available at https://osf.io/n9yqw. No power analysis was conducted, and the studies were not preregistered.

## 2 Method

We conducted an archival content analysis of titles and abstracts published in social and personality psychology and related fields between 1990 and 2020.

### 2.1 Journal Selection

We compiled a list of relevant journals in social psychology by searching SCImago Journal Rank (SJR) for top‐ranked social psychology journals and combing the websites of well‐known publishers in the field (namely, SAGE, Elsevier, Taylor & Francis and Wiley), for a list of 69 journals in social psychology and related fields.^3^

### 2.2 Data Retrieval

Every title and abstract dating from 1990 to 2020 that was publicly available on each journal website was retrieved (online first articles were not included), through web scraping or by manually copying the information. Most abstracts were available and could be accessed.^4^

### 2.3 Data Processing

After excluding errata, corrigenda and editorials from the analysis, the resulting data comprised the titles and abstracts of 78,105 articles from 69 journals (see Figure 1). Considering the distribution of articles in journals, each journal yielded an average of 1131.96 articles (SD = 1460.37, distribution median = 774).

**FIGURE 1:**
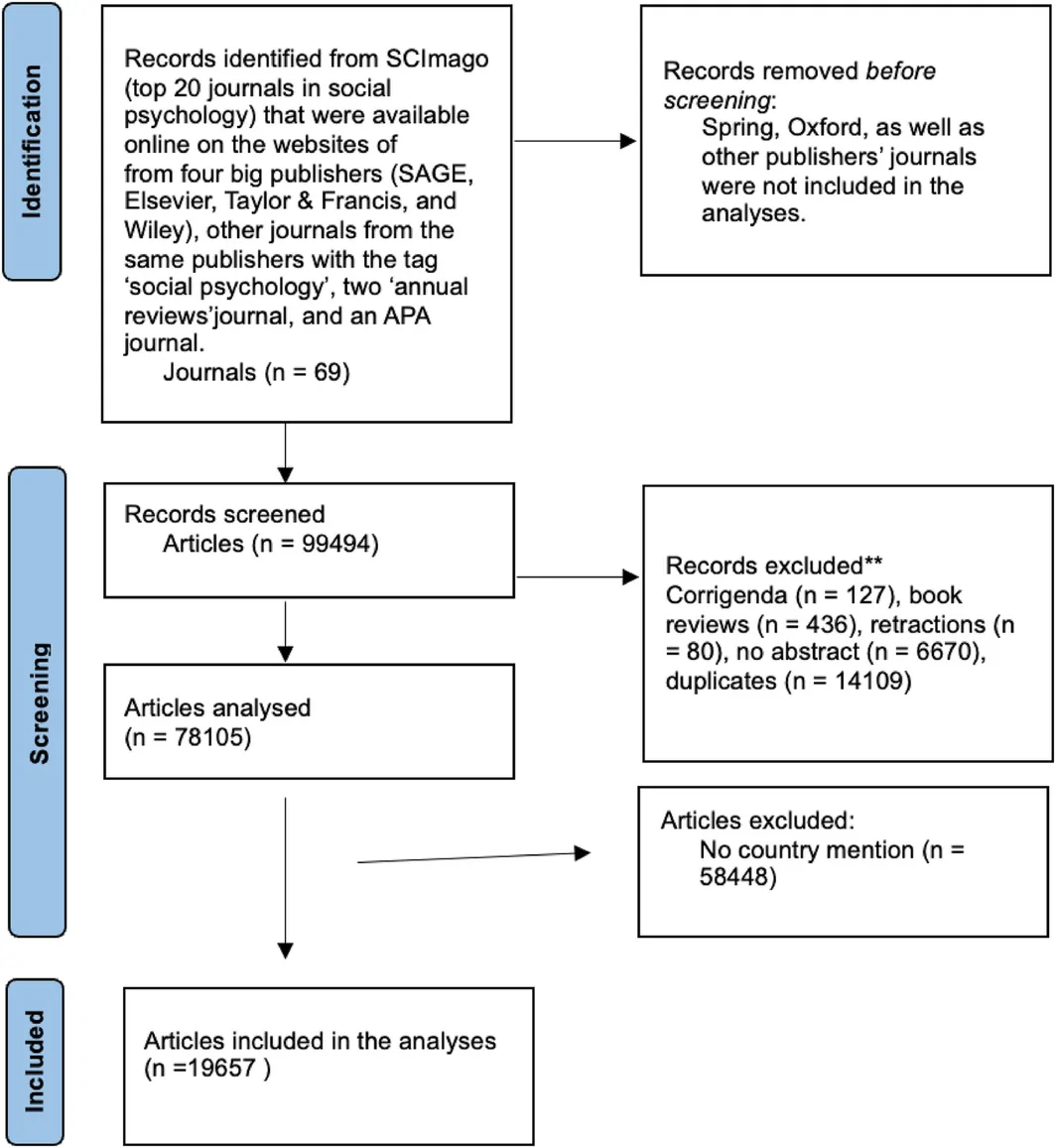
Diagram showing the data processing of the current study.

We counted the frequencies of country names (e.g., ‘United States’, ‘U.S.’) and their peoples (e.g., ‘Argentinian’, ‘Argentine’) using Arnett's (2008) and Thalmayer et al.'s (2021) country classification: Europe, Asia, Latin America (including the Caribbean), Africa, Middle East the United States, Israel and ‘English‐Speaking’ (the United Kingdom, Canada, Australia and New Zealand).^5^

## 3 Results

### 3.1 Cultural Awareness

19,657 articles contained country mentions in their titles or abstracts across the 69 journals (*M* = 284.88, SD = 347.47 per journal; Mdn = 208). Substantial variation was observed between journals, suggesting that mentioning a country in the title or abstract of an article depends on the main theme of the journal.^6^ Roughly half (34) of the journals had country mentions in more than 25% of the retrieved titles and abstracts. Only eight journals had country mentions in more than 50% of the retrieved titles and abstracts, most of them related to culture or localised issues (‘*Asian Journal of Social Psychology*’, ‘*Behavioral Sciences of Terrorism and Political Aggression*’, ‘*Dynamics of Asymmetric Conflict*’, ‘*Hispanic Journal of Behavioral Sciences*’, ‘*International Journal of Intercultural Relations*’, ‘*International Journal of Psychology*’, ‘*Journal of Cross‐Cultural Psychology*’ and ‘*Psychology and Developing Societies*’) (see Table 1). This result suggests that cultural awareness remains low in most journals in the field of social psychology.

**TABLE 1:** Percentage of mentions of each region by total articles by journal.

Journal	United States	English‐speaking	Africa	Asia	Europe	Israel	Latin America	Middle East	Mentions/total
*Annual Review of Organizational Psychology*	100%	—	—	—	—	—	—	—	7/147 (4.8%)
*Annual Review of Psychology*	96.8%	9.7%	—	—	—	—	—	—	31/638 (4.9%)
*Asian Journal of Social Psychology*	22.5%	9.9%	0.3%	89.9%	3.3%	1.6%	0.8%	3.8%	365/619 (59%)
*Behavioral Sciences of Terrorism and Political Aggression*	35%	28%	10%	9%	22%	2%	2%	21%	100/173 (57.8%)
*British Journal of Social Psychology*	18.8%	46.4%	5.3%	9.2%	32.6%	4.3%	3%	9.9%	304/1194 (25.5%)
*Corrections*	80%	10%	5%	10%	—	—	—	—	20/132 (15.2%)
*Culture & Psychology*	24.9%	11.5%	6.3%	28.5%	33.6%	3.2%	9.1%	6.3%	253/734 (34.5%)
*Current Opinion in Psychology*	91.2%	—	1.8%	7%	—	—	1.8%	1.8%	57/973 (5.9%)
*Deviant Behavior*	50.6%	16.2%	2.4%	21.6%	15.9%	1.5%	3.3%	5.1%	334/1301 (25.7%)
*Discourse Studies*	16%	14.3%	4.6%	26.1%	39.1%	4.2%	3.4%	5.5%	238/650 (36.6%)
*Dynamics of Asymmetric Conflict*	31.3%	22.2%	7.1%	26.3%	12.1%	20.2%	3%	38.4%	99/173 (57.2%)
*Emotion Review*	80.9%	2.9%	1.5%	2.9%	17.6%	—	—	—	68/695 (9.8%)
*Emotion, Space and Society*	16.2%	41.4%	5.7%	14.8%	25.7%	2.4%	11.4%	5.2%	210/475 (44.2%)
*European Journal of Personality*	27%	10.5%	0.8%	13.9%	69.6%	1.7%	2.1%	2.5%	237/1048 (22.6%)
*European Journal of Social Psychology*	26.7%	22.4%	3.7%	11.2%	49.8%	6%	3.4%	11.4%	464/2004 (23.2%)
*Family Science*	23%	13.1%	1.6%	13.1%	60.7%	4.9%	4.9%	9.8%	61/142 (43%)
*Group Analysis*	25.7%	22.3%	4.1%	2.7%	48%	10.1%	4.1%	7.4%	148/847 (17.5%)
*Group Processes & Intergroup Relations*	40.8%	16.9%	2.4%	12.2%	32.2%	7.5%	4.7%	13.7%	255/956 (26.7%)
*Hispanic Journal of Behavioral Sciences*	74.5%	1%	—	0.8%	19.5%	0.2%	70.2%	—	604/910 (66.4%)
*Intelligence*	31.6%	20.2%	7.1%	12.8%	43.3%	1.4%	5.2%	3.3%	367/1517 (24.2%)
*International Journal of Intercultural Relations*	30.1%	18.8%	7.4%	36.3%	30%	8.6%	6.3%	10.6%	962/1364 (70.5%)
*International Journal of Psychology*	19.5%	12.3%	8.7%	44.6%	30.8%	4%	7.3%	10.3%	917/1497 (61.3%)
*International Journal of Transgenderism*	44.2%	27.9%	—	16.3%	23.1%	—	7.7%	4.8%	104/289 (36%)
*Japanese Psychological Research*	16.4%	3.8%	0.5%	88.5%	2.7%	0.5%	—	—	183/712 (25.7%)
*Journal for the Theory of Social Behavior*	50.7%	20.9%	3%	25.4%	22.4%	3%	3%	3%	67/929 (7.2%)
*Journal of Applied Social Psychology*	41.8%	19.2%	2.6%	17.8%	27%	4.8%	5%	4.5%	625/3622 (17.3%)
*Journal of Behavioral Decision Making*	57.1%	35.7%	—	7.1%	28.6%	7.1%	—	—	14/209 (6.7%)
*Journal of Bisexuality*	43.3%	37.3%	4.5%	5.2%	19.4%	0.7%	3%	2.2%	134/557 (24.1%)
*Journal of Community & Applied Social Psychology*	13.9%	42.7%	7.6%	10.1%	30.9%	1.5%	6.3%	4.6%	475/965 (49.2%)
*Journal of Criminal Justice*	63.3%	16.5%	2.8%	11.4%	8.6%	1.6%	3.9%	1.6%	431/1584 (27.2%)
*Journal of Cross‐Cultural Psychology*	46.4%	21.8%	8.9%	52.1%	28.9%	6.6%	10.5%	8.7%	1237/1625 (76.1%)
*Journal of Experimental Social Psychology*	68.7%	10.4%	2.5%	18.4%	15.8%	5.4%	2.2%	9.2%	316/2750 (11.5%)
*Journal of Family Communication*	71.9%	6.2%	3.1%	31.2%	7.8%	3.1%	6.2%	4.7%	64/341 (18.8%)
*Journal of GLBT Family Studies*	44.6%	26.6%	4.3%	7.9%	20.1%	4.3%	0.7%	0.7%	139/379 (36.7%)
*Journal of Health and Social Behavior*	75.7%	9.2%	3.2%	10.8%	6.5%	—	11.4%	—	185/513 (36.1%)
*Journal of Homosexuality*	35.7%	18.6%	3.8%	13.9%	26%	2.4%	6.5%	3.4%	703/2033 (34.6%)
*Journal of LGBT Youth*	41.3%	35.5%	7.2%	6.5%	12.3%	4.3%	6.5%	0.7%	138/378 (36.5%)
*Journal of Language and Social Psychology*	34.7%	31%	3.8%	24.4%	31.5%	1.4%	0.5%	4.7%	213/774 (27.5%)
*Journal of Lesbian Studies*	41.2%	28.6%	4.5%	17.1%	18.1%	2%	5%	2.5%	199/787 (25.3%)
*Journal of Occupational Science*	20.8%	47.9%	6%	10.6%	23.8%	1.1%	6.8%	3%	265/714 (37.1%)
*Journal of Occupational and Social Psychology*	14.3%	29.9%	4.5%	19.5%	37.7%	3.9%	0.6%	1.3%	154/756 (20.4%)
*Journal of Organizational Behavior*	27.1%	15.8%	2.2%	38.8%	25.6%	9.5%	2.2%	3.3%	273/1643 (16.6%)
*Journal of Personality and Social Psychology*	64.8%	11.3%	2.7%	24.7%	22.2%	5.1%	4%	3.3%	603/4757 (12.7%)
*Journal of Research in Crime and Delinquency*	51.1%	19.1%	0.7%	14.2%	18.4%	3.5%	5%	1.4%	141/630 (22.4%)
*Journal of Research in Personality*	43.3%	13.8%	1.7%	20.5%	30.6%	3.7%	6.2%	4.5%	356/2103 (16.9%)
*Journal of Sexual Aggression*	21.6%	64.2%	3.7%	3.7%	17.2%	0.7%	1.5%	0.7%	134/494 (27.1%)
*Journal of Social Issues*	56.4%	15.6%	7%	13.3%	16.7%	5.1%	6.6%	6.6%	473/1431 (33.1%)
*Journal of Social and Personal Relationships*	33.7%	11.2%	0.8%	25.7%	24.1%	9.2%	8%	3.6%	249/1733 (14.4%)
*Journal of Theoretical Social Psychology*	50%	37.5%	—	25%	12.5%	—	—	12.5%	8/44 (18.2%)
*Learning, Culture and Social Interaction*	11.9%	11.1%	4.8%	17.5%	50%	1.6%	7.9%	7.9%	126/322 (39.1%)
*Organizational Psychology Review*	100%	—	—	—	—	—	—	—	5/135 (3.7%)
*Personality and Individual Differences*	22.7%	21%	2.3%	24.1%	38.1%	2.6%	2.4%	4.6%	2340/10,235 (22.9%)
*Personality and Social Psychology Bulletin*	65.7%	20%	1.9%	25.4%	17.1%	7.2%	2.3%	8.5%	469/3573 (13.1%)
*Personality and Social Psychology Review*	87.2%	7.7%	5.1%	10.3%	2.6%	2.6%	2.6%	2.6%	39/460 (8.5%)
*Personnel Psychology*	63.4%	14.1%	1.4%	33.8%	8.5%	1.4%	1.4%	7%	71/819 (8.7%)
*Political Psychology*	43.3%	17.6%	2.9%	10.1%	31.1%	8.9%	2.7%	8.7%	517/1092 (47.3%)
*PsyCh Journal*	11.2%	10.1%	—	80.9%	14.6%	1.1%	1.1%	2.2%	89/299 (29.8%)
*Psychological Science*	61.4%	12%	3.8%	17.6%	24.2%	2.4%	2.6%	3.5%	425/4135 (10.3%)
*Psychological Science in the Public Interest*	87.5%	—	—	12.5%	—	—	—	12.5%	8/47 (17%)
*Psychology and Developing Societies*	12.4%	10.4%	16%	66%	6.4%	2%	7.2%	3.2%	250/363 (68.9%)
*Psychology and Sexuality*	30.8%	38.5%	6.7%	5.8%	26.9%	1.9%	4.8%	3.8%	104/294 (35.4%)
*Psychology, Crime & Law*	18.9%	40.2%	2.3%	8.1%	40.5%	0.4%	0.4%	0.4%	259/1065 (24.3%)
*Religion, Brain & Behavior*	19.5%	26.8%	9.8%	14.6%	12.2%	—	9.8%	—	41/150 (27.3%)
*Social Development*	44.4%	12.5%	3.2%	30.1%	20.4%	1.9%	4.6%	5.1%	216/1101 (19.6%)
*Social Issue and Policy Review*	86.7%	6.7%	—	—	—	13.3%	—	13.3%	15/107 (14%)
*Social Psychological and Personality Science*	71.2%	9.6%	1.9%	18.3%	17.3%	5.8%	3.8%	9.6%	208/957 (21.7%)
*Social Psychology Quarterly*	68.2%	3.5%	4.7%	20%	23.5%	2.4%	2.4%	5.9%	85/345 (24.6%)
*Social and Personality Psychological Compass*	85.3%	21.1%	5.5%	13.8%	11%	1.8%	4.6%	3.7%	109/900 (12.1%)
*Journal of Forensic Psychiatry & Psychology (UK)*	16.8%	57.2%	2.4%	5.7%	24.9%	0.7%	0.7%	1.7%	297/892 (33.3%)

*Note:* ‘Mentions/total’ indicates the absolute number of analysed articles that mention at least one country, the total number of analysed articles and its percentage, respectively. Each region's percentage is calculated relative to the total number of articles mentioning countries of each journal. Thus, the sum of percentages of all regions may exceed 100% if articles mention more than one country.

### 3.2 Cultural Diversity

In 41 journals, the most mentioned region was the United States, whereas 11 journals mentioned Europe the most, eight journals referenced ‘English‐Speaking’ regions most often, eight journals referenced Asia the most, and one journal mentioned the Middle East the most. The other regions were not the most mentioned in any journal.

The United States was the most mentioned region overall, followed by English‐speaking and Europe. Asia follows the Western regions, followed by Israel. Latin America and the Middle East have a very low frequency in most journals, except for those related to local issues concerning these regions. Lastly, Africa was seldom mentioned.

### 3.3 Change Over Time

We conducted a series of regressions to investigate how cultural awareness changed over time. Firstly, we conducted a mixed effects regression (random slopes) with year as the independent variable, and proportion of country mention (total mentions/total number of articles) as the dependent variable, and years nested within journals as the random effect (ICC = 0.77) (formula: proportion of mentions ˜ year). The model's total explanatory power was substantial (conditional *R* ^2^ = 0.78; marginal *R* ^2^ = 0.07). The model's intercept, corresponding to year = 0 (1990), was at 0.81 (95% CI [0.34, 1.28], *t*(723) = 3.40, *p* < 0.001). The effect of year was statistically significant and positive (beta = 0.05, 95% CI [0.03, 0.07], *t*(723) = 4.92, *p* < 0.001; Std. beta = 0.27, 95% CI [0.16, 0.38]).

Secondly, we investigated whether the proportions of the United States, other‐WEIRD (Europe and English‐speaking), and Majority World (Asia, Latin America, Middle East, Africa) changed with time to grasp how diversity changed. For this, we fitted three logistic mixed models with proportion of region mention (region mentions/total number of articles) as the dependent variable, and years nested within journals as the random effect to investigate how the proportions of United States, other‐WEIRD and Majority World mentions changed over time. Over the 30‐year period (1990–2020), proportions of US mentions declined significantly (beta = −0.015, 95% CI [−0.022, −0.008], *p* < 0.001), while proportions of other‐WEIRD did not vary significantly (beta = 0.003, 95% CI [−0.004, 0.010], *p* = 0.394) and Majority World increased significantly (beta = 0.042, 95% CI [0.035, 0.049], *p* < 0.001) increased.

In short, mentions of countries increased over time (1990–2020), indicating an increase in awareness of the importance of culture. While there is a slight, almost imperceptible decrease in terms related to the United States compared to terms related to other countries, while mentions of other‐WEIRD and Majority World countries remain are slightly increasing (see Figure 2). Notably, the figure shows that mentions of the Majority World countries are comparable to mentions of the United States alone, while mentions of other‐WEIRD countries consistently exceed those of the Majority World, with the only exception being 1990.

**FIGURE 2:**
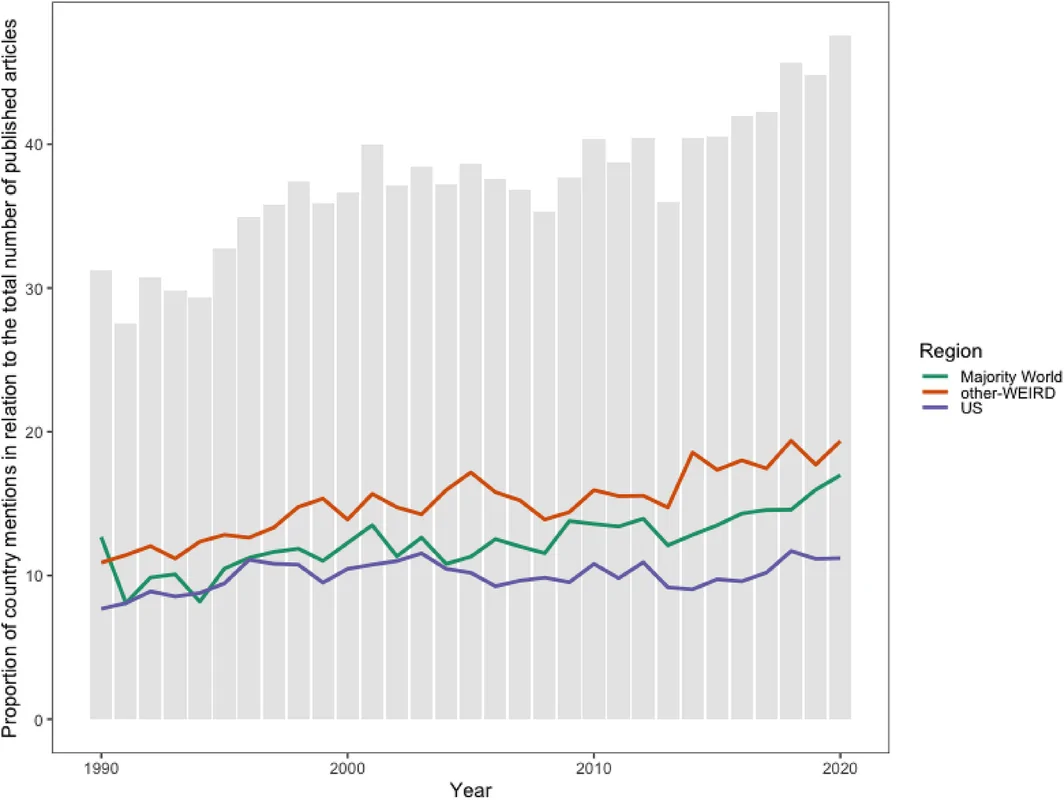
Proportion of total country mentions and per region in relation to the total published articles. Grey bars represent the percentage of articles containing at least one country mention per year. Coloured lines represent the proportion of each region (United States, other‐WEIRD or Majority World) in the articles that have at least one country mention. For this analysis, only journals containing country mentions in at least 10% of their articles were included (*N*_Journals_ = 60).

## 4 Discussion

The goal of this research project was to analyse cultural awareness in social psychology. We argue that it is only by taking culture into consideration that studies can properly contribute to the field through theory development and empirical evidence. In other words, a study that does not present and discuss the cultural context in which it was conducted has unclear boundary conditions. The results show that country mentions were more common in journals focused on culture, international studies, and local issues, and less common in journals focused on reviews, theory and business research. Thus, cultural awareness is influenced by research themes.

The present research also confirmed findings from previous research, such as cultural awareness being disproportional across regions of the world, and WEIRD authors having a tendency to omit country‐level information while Majority World authors being more likely to include country‐level information in their titles and abstracts^7^ (Cheon et al. 2020; Kahalon et al. 2022; Rad et al. 2018; Torres and Alburez‐Gutierrez 2022).

Our results suggest a general increase in awareness related to the importance of culture over time. Compared to 1990, more articles have country‐level information in their titles and abstracts across journals in different genres of social psychology. Moreover, there appears to be a gradual decrease in the proportion of mentions of the United States, and an increase of Majority World over time. These observed tendencies are gradual; the United States and other‐WEIRD countries are still dominant in social psychology in terms of mentions.

### 4.1 Implications

The results presented here can serve as a resource for researchers to reflect on journals, their primary themes and the importance given to culture. While cultural awareness may seem obvious for researchers studying local issues or culture, it is equally important in other subfields, albeit not as commonly mentioned. For example, in the current age of globalisation, businesses have to adjust culturally to diverse locations; thus, scholars are supposed to pay attention to and be sensitive to culture, but that does not seem to be the case. Furthermore, in reviews, meta‐analyses and general theory development, it is critical to raise cultural awareness because the predominance of studies conducted on non‐diverse samples directly impacts theory development and introduces bias into reviews and meta‐analyses. If culture is not integrated as a central factor, generalisability of psychology remains an impossibility.

### 4.2 Recommendations

We recommend that authors mention country in their titles and abstracts, as this is greatly informative for readers, especially those outside of the WEIRD circuit. To put it simply, the more contextual information is displayed in titles and abstracts, the easier it is for the reader to understand if the research results can be applied to their reality. Similarly, we recommend journals, editors and reviewers to be welcoming to authors who disclose as much contextual information about their sample as possible.

Beyond these recommendations, we believe that structural changes are needed to solve this issue. First, details about the sample, such as country, age, gender and ethnicity, should be included in the metadata of each article stored on a journal's webpage. Journals are mostly digital nowadays, and including more information in the metadata may be costly to set up initially, but will be significantly beneficial for the whole field in the future. This could also serve as a convenient way to oblige researchers who are reluctant to include country information in their titles and abstracts. Similarly, authors' affiliation should be highlighted, quantified and displayed to show authorship diversity.

Second, the review process needs to change to promote and accommodate research with diverse samples and authors from the Majority World. The current review process adopted in most journals favours affluent researchers with access to large samples, expensive research materials, cutting‐edge technology and linguistic resources (e.g., English native speakers). Editors and reviewers often reject articles from the Majority World based on the characteristics of researchers that shows a lack of such resources. This needs to change in favour of an evaluation based on scientific rigour and soundness rather than research resources.

Third, we encourage big team science (BTS), which can be a powerful tool for increasing diversity in the field. However, this needs to be done in a way that researchers from various parts of the world are equally responsible for the studies and theories developed (Jia 2026b; Vaidis et al. 2024).

Fourth, we support and endorse the recommendations made by other researchers invested in the same issue (see supplemental materials for a summary of those recommendations and a list of references).

### 4.3 Limitations and Future Directions

Our studies only analysed journals in English that follow the American Psychological Association (APA) guidelines, which arguably might be biased towards a more ‘Americanized’ (or WASP) way of doing psychological research. Analyses of journals published in other languages could provide a global view of how diversity is portrayed in psychological research worldwide.

Psychologists in the Global South do not always subscribe to the WASP; in many countries, psychologists focus on local issues and publish in languages other than English (Adair et al. 2002) journals, that is, published in other languages, as well as other forms of publications, such as books and conference proceedings, were not included in the analysis. An analysis of journals outside the WASP would be interesting.

To count occurrences, we looked for terms that refer to countries and demonyms, excluding other ways of referring to culture, such as cities or regions. Thus, cross‐cultural studies with many countries containing sentences such as ‘a cross‐cultural study in 30 countries’ or ‘a comparison between individualist and collectivist populations’ were excluded from the analysis. Accordingly, we focused on country‐level diversity, as it is a commonly reported difference in cultural, cross‐cultural and social psychology. Despite national culture exerting a major influence on people's psyche (Akaliyski et al. 2021), cultural diversity extends beyond national affiliations.

## Author Contributions

**Igor de Almeida:** conceptualization, investigation, writing – original draft, methodology, validation, visualization, writing – review and editing, formal analysis, data curation, project administration. **Yukiko Uchida:** conceptualization, writing – original draft, writing – review and editing, supervision, project administration. **Kongmeng Liew:** supervision, writing – review and editing, data curation. **Lean Franzl Lim Yao:** writing – review and editing, data curation, formal analysis. **Eiji Aramaki:** supervision.

## Funding

The authors have nothing to report.

## Ethics Statement

The authors have nothing to report.

## Conflicts of Interest

The authors declare no conflicts of interest.

## Supporting information


**Table S1:** Total number and percentage of authors mentioning their affiliation country in titles and abstracts.
**Table S2.1:** Comparison of the present study analyses to previous research.
**Table S2.2:** Comparison to (Arnett 2008) data from the *Journal of Personality and Social Psychology*.
**Table S2.3:** Comparison to (Thalmayer et al. 2021) data from the *Journal of Personality and Social Psychology*.
**Table S2.4:** Comparison to (Rad et al. 2018) data from the *Psychological Science* journal.
**Table S3.1:** Topics classified from the data from Study S1.
**Table S3.2:** Residual analysis.
**Table S4:** Percentage of mentions of each region by total articles by journal.

## Data Availability

Processed data and analysis code are available at: https://osf.io/n9yqw/.

## Endnotes

^1^
Culture as well as context can be understood in many levels and various ways. In the present manuscript, we use the word culture as an abbreviation of ‘national culture’, or simply the culture of a country. Similarly, we use the word context as an abbreviation of ‘national cultural context’.

^2^
In this manuscript, social psychology is used to mean social and personality psychology, as the fields of social psychology and personality psychology are commonly found together.

^3^
Although some journals do not specifically mention social psychology in their titles, all of them are classified as social psychology journals in journal rankings or in the publisher webpage, and they publish research from this subfield.

^4^
Data were retrieved in June of 2021. Eighty‐nine articles did not have an abstract available on the website. Also, broken weblinks and some other technical issues sometimes hindered data retrieval.

^5^
These categories are mutually exclusive. For instance, the United Kingdom is only included in ‘English‐Speaking’, not in ‘Europe’.

^6^
A topic model was conducted to investigate the likelihood of articles with different themes having country mentions in their titles and abstract. See Supporting Information for details.

^7^
See Supporting Information for additional analyses related to this matter.
