# Supplementary material for: To Whom National Culture Matters: An Analysis of Mentions of Countries in Titles and Abstracts in Social and Personality Psychology and Related Fields From 1990 to 2020
Source: Int J Psychol. 2026 Oct 2;61(6):e70275. doi: 10.1002/ijop.70275 (PMC13632611; doi:10.1002/ijop.70275)
Supplement: Supplementary file 1 [file IJOP-61-e70275-s001.pdf]

Supplemental materials - To whom culture matters: an analysis of mentions of countries in titles and abstracts in social psychology and related fields from 1990 to 2020

About the statistical analyses

All analyses were done using the R programming language version 4.2.3 (R Development Core Team, 2023)

Graphs were made using the packages ggplot2 version 3.4.2(Hadley Wickham, 2016) and egg 0.4.5 (Augie, 2017).

Multilevel analyses were done using the packages multilevel 2.7(Bliese, 2012) and LmerTest 3.1-3 (Kuznetsova et al., 2022).

Expanding the discussion

The lack of cultural diversity in psychological research has been widely discussed for several decades. Numerous researchers have identified this problem, examined its underlying causes, and proposed ways to improve the current state of the field.

Due to word limits and restrictions on the number of references, it was not possible to include all previous studies supporting our argument. Therefore, we provide a slightly expanded discussion here.

Our intention with this expanded discussion is not to provide an exhaustive list of previous publications related to the main topic of the manuscript, rather to further contextualise the issue, give support to it, and provide more references to the reader who is interested in this topic. In other words, we briefly included here references that we planned to include in the main manuscript but unfortunately could not do.

Diagnosing the lack of diversity in psychological research

We focus on the subfield of social and personality psychology in our project. However, researchers across multiple subfields of psychology have shown and argued how neglecting the importance of culture compromises the quality of research outcomes. Representative examples emerge from organizational psychology (Pitesa & Gelfand, 2023; Schimmelpfennig et al., 2025), developmental psychology (Nielsen et al., 2017), evolutionary psychology (Pollet & Saxton, 2019), psychotherapy research (Solomonov et al., 2025), music psychology (Jakubowski et al., 2025), cognitive neuroscience (Dotson & Duarte, 2020), and the teaching of psychological science (Richmond et al., 2015). This issue can be related to the lack of diversity in editorial boards (Arnett, 2008; Hafeez et al., 2019; Palser et al., 2022; Thalmayer et al., 2020).

In social and personality psychology, previous research has shown that the current model of research, aiming universality, is not effective and actually only explains one specific culture (the one in which the knowledge was developed), and fails in recognising it (Henrich et al., 2010; Hornstein & Star, 1990; Pepitone & Triandis, 1987; Rozin, 2001).

In (cross) cultural psychology, another problem emerged. While researchers in 'classical' social psychology conduct experiments with Western (mostly US) participants and use the data to explain the whole humanity, (cross) cultural psychology, often considered a subfield of social psychology, researchers collect data from East Asian countries and use it to explain all cultures that aren't Western (Krys et al., 2022; Uskul & Mesquita, 2026; Vignoles, 2018).

Importantly, there are also voices among the psychological research community that do not necessarily support our argument or point to the same direction. There are scholars arguing that (WASP) psychology is actually becoming more international (Webster et al., 2009, 2021). Other scholars argue that studies should not necessarily have external validity, e.g. should not be worried about universality (Mook, 1983), and that, if conducted properly and interpreted

adequately, studies on local population and convenient samples, regardless of being WEIRD or not, are previously valuable to psychological literature (Sherman, 2025). Another point of view is that diversity is not necessary for the development and testing of theories (Stroebe, 2024). There is also those authors who criticise research on the lack of diversity as being more political than scientific (Hommel, 2024a, 2024b).

Issues hindering diversity in mainstream psychological research

Mainstream psychology (or WASP), as well as other sciences, tends to ignore publications that aren't available in English. Editorial boards of influential consist of mainly native English speakers. Non-native English speakers have their manuscripts rejected more often than native speakers, and they are often required to replicate Western-made psychological findings with their local samples in order to be considered a proper researcher (Tindle, 2021). This not only hinders many researchers' career development, but also harms the development of knowledge in general. Researchers outside the western world have to either focus on local issues and face severe consequences of not being connected with "the globalized world", or embrace the mainstream scientific community based in English and largely ignore locally produced knowledge, creating severe biases in their research (Amano et al., 2016).

Another issue is that psychological research has not engaged properly with neurodivergent populations, labelling them as abnormal, deficient, dysfunctional, or impaired. The inclusion of those populations in psychology as neurological variations (Manalili et al., 2023) and with diverse epistemologies about neurodivergence (Nair et al., 2026) may benefit the whole field providing new theories and points of view.

Recommendations to increase cultural awareness and cultural diversity in research

As stated in the main manuscript, we support and endorse ideas and initiatives that aim to increase diversity and cultural awareness in the field.

Big Team Science (BTS) is one approach that can help increase both cultural awareness and cultural diversity in research. It is defined as "a large-scale scientific project that is built upon collective effort, undertaken due to the complexity of the scientific issues involved, and facilitated by a distribution of tasks and specialties within the team" (Vaidis et al., 2024). It has various advantages and tackles directly issues related to replicability, diversity, and statistical power. However, it comes with many difficulties such as differences in law, practices, epistemologies, tendencies, and communication across countries (Vaidis et al., 2024).

Puthillam et al (2024) conducted a hackathon at a psychological conference to develop guidelines to improve diversity in psychological research. In their paper, they provided a detailed list of proposals that could help improving psychological research from dissemination of research outcomes to how researchers can work on their research design and analysis, passing though changes in the review and publication process. Similarly, Aly et al (2023)propose a deep change in the peer review culture.

Other researchers encourage big team science as a way to improve diversity in the field. Some with detailed guidelines on how to build up teams (Baumgartner et al., 2023), discussions about how it improves multicultural research (Lui et al., 2022), and reflections about its limitations (Ghai et al., 2024).

Finally, there are researchers who propose creating more spaces for researchers outside the Western world to share their research (Krys et al., 2026), and that simply having non-Western sample should be considered of great interest (Rad et al., 2018).

Study S1 – Are Majority world researchers more likely to include country-level information in their titles and abstracts?

Previous studies have shown that majority world authors are more likely to include country level information in their titles and abstracts than authors from WEIRD cultures (Castro Torres & Alburez-Gutierrez, 2022; Cheon et al., 2020; Kahalon et al., 2022).

In this study we tried to replicate these findings using our data.

Method

Since the data used in Study 1a did not contain information about author's affiliation, i.e., in which country their institution is located, data from PubMed was retrieved. Whilst the goal was to retrieve data from the same journals and during the same span as in Study 1a, only 18449 articles from 62 journals could be retrieved, since not every journal sampled in the main study was indexed in PubMed and not all articles were available on the platform.

Data treatment and processing was the same as in the main study.

Results

As suggested by previous research, the results of this study suggest a difference in cultural awareness across world regions. Authors affiliated with institutions in Asia or Africa mentioned their country in about 40% of titles and abstracts of the articles analyzed, whereas only 12,4% of researchers from U.S. institutions and 13.4% of researchers from institutions in English-speaking countries did so (see Table S1). To compare the likelihood of a country mention in titles and abstracts among researchers based in different regions, we conducted a logistic regression with author's affiliation (location of the first author's institution) as the predictor variable, and mention of their country (0 = no mention, 1 = mention) as the outcome variable. Results indicate that there was a significant association between region (using the U.S. as the baseline) and country mention (Wald $\chi2 = 3865.8$, $p < .001$). For the regression,

U.S. based researchers were used as the baseline (intercept, OR = 0.14, 95% CI [.13, .15], p<.001). Researchers in Africa (OR = 4.90, 95% CI [3.31, 7.18], p < .001), Asia (OR = 4.68, 95% CI [4.04, 5.40] , p<.001), Europe (OR = 1.82, 95% CI [1.62, 2.05] , p<.001), Israel (OR = 2.34, 95% CI [1.73, 3.11] , p<.001), Latin America (OR = 4.86, 95% CI [3.33, 7.03] , p<.001), and the Middle East (OR = 12.06, 95% CI [8.05, 18.34] , p<.001) were significantly more likely to mention their country in titles and abstracts than researchers in the U.S. Researchers in English-speaking countries (OR = 1.10, 95% CI [0.97, 1.25], p = .13) were just as likely as US based researchers to mention their country in titles and abstracts[1]. These results show that researchers in the US and English-speaking countries are significantly less likely to mention their countries of affiliation in titles and abstracts compared to researchers in other regions. Additionally, the findings suggest that the likelihood of mentioning one's affiliation country may be influenced by the country's cultural proximity to the US. While researchers in Israel and Europe do differ significantly from researchers in the U.S., their odds ratio are not as pronounced as those in Asians, Latin Americans or Middle Easterners institutions (see Atari et al (2023) for a discussion on the WEIRDness continuum).

Table S1

Total number and percentage of authors mentioning their affiliation country in titles and abstracts

Country mention	Africa	Asia	English-speaking	Europe	Israel	Latin America Caribbean	Middle East	United States
No	65 (59.09%)	581 (60.58%)	2400 (86.55%)	1962 (79.63%)	191 (75.2%)	96 (66.67%)	37 (37%)	8153 (87.63%)

[1] A Chi-squared test yielded similar results (X^2(7, N = 16057) = 780.73, p < .001, V = .22).

Country mention	Africa	Asia	English-speaking	Europe	Israel	Latin America Caribbean	Middle East	United States
Yes	45 (40.91%)	378 (39.42%)	373 (13.45%)	502 (20.37%)	63 (24.8%)	48 (33.33%)	63 (63%)	1151 (12.37%)

Study S2 – Do country-level mentions in titles and abstract reflect diversity in sample?

As discussed throughout our manuscript, a country-mention in titles or abstracts reflects the intention of the authors to include such information in what will be the first contact a reader has with their research. One can assume that most likely this information will reflect the sample studied in the studies contained in the manuscript, however there are two issues with this assumption – 1) a country mention can be used to contextualize a societal issue, provide historical context, indicate an adaptation, increase niche searchability, etc; 2) following previous studies and study S1, country-level information in titles and abstracts may be more common for majority world samples and less common for WEIRD samples, producing misleading results.

To investigate how it works in practice, we compared our data to Arnett, Thalmayer and Rad ‘s studies, since our data overlaps with theirs. Specifically, we compared our results to two analyses of the Journal of Personality and Social Psychology: Arnett's (2009) analysis from 2003 to 2007, as well as Thalmayer and colleagues' (2020) analysis from 2014 to 2018. Additionally, we compared our results to the analysis of Psychological Science by Rad and colleagues' (2018) of 2014 and 2017 (see Table S2.1 for a summary; and Tables S2.2, S2.3 and S2.4 for the values used in the statistical tests).

Their analyses followed a specific pattern that was reflected in our analyses: the US is the most mentioned country, followed by Europe, English Speaking countries, Asia, a few mentions of Africa, Israel and Latin America, and rare mentions of the Middle East. However, when comparing the proportions of country-level mentions in titles and abstracts versus sample descriptions, we noticed that WEIRD samples were less commonly mentioned in titles and abstracts, while countries from the Majority World were inflated in our results. To investigate this specific issue, we conducted three statistical tests.

Significant relationships were observed in the three comparisons: US and other WEIRD (English-speaking, European) samples were less likely to be mentioned in titles and abstracts, while Majority World (African, Asian, Latin American, and Middle Eastern) were more likely to be mentioned in titles and abstracts in JPSP in the 2003-2007 period ($X^2(2, N = 848) = 87.00$, $p < .001$, $V = .32$), as well as in the 2014-2018 period ($X^2(2, N = 791) = 41.88$, $p < .001$, $V = .23$), and in Psychological Science in 2014 and 2017 (issues 10~12) (Fisher's Exact Test, $p < .001$).

Table S2.1

Comparison of the present study analyses to previous research

Journal	Period	Analysis	USA	Asia	Eng-speaking	Europe	Africa	Latin America	Middle East	Israel
Journal of Personality and Social Psychology	2003-2007	Arnett (2008)	62%	4%	12%	19%	1%	1%	0%	2%
		Present Study	41.4%	22.8%	6.2%	20%	1.4%	2.8%	2.8%	2.8%
	2014 – 2018	Thalmayer et al (2020)	60%	6%	11%	17%	1%	1%	1%	3%
		Present Study	38.9%	20.6%	6.9%	22.9%	-	1.5%	3.8%	3.8%
Psychological Science	2014 and 2017*	Rad et al (2018)	58.3%	5.1%	13.1%	16.7%	1.3%	-	-	4.9%
		Present Study	51.2%	11.6%	4.7%	14%	7%	2.3%	7%	2.3%

Note. Arnett (2008), Thalmayer et al (2020) and Rad et al (2018) analyzed full texts from the journals and coded the description of the samples written in the articles. Their percentages refer to the total samples published in each journal during the mentioned period; the percentages for the present study refer to the total of titles and abstracts that contained at least one country mention. * Volumes 10,11 and 12.

Table S2.2
Comparison to (Arnett, 2008) data from the Journal of Personality and Social Psychology

region			
Dataset	Non-WEIRD	Other-WEIRD	US
Current study	43	38	60
Arnett (2003 and 2007)	38	219	450

Table S2.3
Comparison to (Thalmayer et al., 2020) data from the Journal of Personality and Social Psychology

Dataset	Non-WEIRD	Other-WEIRD	US
Current study	35	39	51
Thalmayer et al (2014 and 2018)	58	192	416

Table S2.4
Comparison to (Rad et al, 2018) data from the Psychological Science journal

Dataset	Non-WEIRD	Other-WEIRD	US
Current study	8	22	
Rad et al. (2014 and 2017)	33	141	275

Study S3 – Are research topics related to the likeliness of a country-level mention?

We conducted a topic model analysis to investigate whether some research topics are more likely than others to have a country-level mention in titles and abstracts.

We fitted the titles of study S1 (N = 18449) into a topic model, 6375 titles were classified in 25 topics. We used the BERTopic library (Grootendorst, 2022) in Python programming language (Van Rossum & Drake, 2011). Despite the downside of having many texts not being classified into a topic, this method was used because it still outperforms other topic modelling algorithms such as LDA and structural topic modeling (Egger & Yu, 2022). See Table S3.1 for the results of the topic model analysis.

Table S3.1
Topics classified from the data from Study S1

Topic	Yes	No	Total	Words
1	12 (1.8%)	655 (98.2%)	667	social, effects, effect, behavior
2	113 (14.6%)	661 (85.4%)	774	visual, attention, search, objects
3	15 (9.3%)	147 (90.7%)	162	intergroup, group, stereotype, ingroup
4	22 (8.5%)	238 (91.5%)	260	infants, children, childrens, young
5	26 (5.4%)	458 (94.6%)	484	language, speech, reading, word
6	65 (8%)	748 (92%)	813	memory, memories, forgetting, working
7	11 (9%)	111 (91%)	122	personality, big, traits, development
8	22 (4.3%)	485 (95.7%)	507	mood, emotion, anger, regulation
9	2 (1.5%)	134 (98.5%)	136	goal, goals, selfcontrol, achievement
10	24 (11.4%)	186 (88.6%)	210	relationships, romantic, relationship, close
11	6 (3.3%)	177 (96.7%)	183	moral, shame, disgust, guilt
12	1 (0.8%)	117 (99.2%)	118	coping, stress, depression, depressive
13	57 (26.1%)	161 (73.9%)	218	sexual, mate, sex, mating
14	16 (6.4%)	234 (93.6%)	250	racial, race, interracial, black
15	18 (23.1%)	60 (76.9%)	78	aggression, power, aggressive, violence
16	1 (1.2%)	84 (98.8%)	85	wellbeing, subjective, satisfaction, income
17	23 (6.7%)	320 (93.3%)	343	gender, sexism, benevolent, women
18	5 (7%)	66 (93%)	71	facial, attractiveness, expressions, emotion
19	32 (10.5%)	272 (89.5%)	304	attachment, adult, styles, security
20	3 (7.9%)	35 (92.1%)	38	selfesteem, acceptance, global, sociometer
21	26 (15.5%)	142 (84.5%)	168	support, stress, loneliness, social
22	10 (7.4%)	126 (92.6%)	136	terror, management, salience, mortality
23	6 (7.9%)	70 (92.1%)	76	attitude, attitudes, implicit, taking
24	1 (1.1%)	89 (98.9%)	90	emotion, emotional, emotions, affective
25	7 (8.5%)	75 (91.5%)	82	marital, marriage, satisfaction, couples

Note.

We conducted a chi-squared test to check whether some themes are more likely than others to have a country mention in an abstract or title (See Table S3.2). Before conducting the chi-squared test, we excluded topics that had 5 or fewer country mentions (topics 8, 11, 15, 17,19, and 23). Results were significant (X^2 (18, N = 5837) = 219.46, p < .001,), showing that there is a relationship between theme and country mention, but with a small effect size (V = 0.19, 95% CI [0.15, 1.00]). Analysis of residuals show that topics 2, 13 and 15 are more likely to have country mentions, topics 1, 5, and 8 are less likely to have country mentions, and topics 3,4,5,6,7,10, 14, 17, 21, 22, 23, and 25 have the expected proportion of country mentions (see Table S3.2).

Although some topics seem to be biased towards having more or having fewer country mentions, most topics have a similar proportion of country mentions.

Table S3.2
Residual analysis

Topic	Residuals (country mention / no country mention)	p values (country mention / no country mention)
1	-6.753 / 6.753	<.001 / <.001
2	6.178 / -6.178	<.001 / <.001
3	0.231 / -0.231	1 / 1
4	-0.171 / 0.171	1 / 1
5	-2.749 / 2.749	0.227 / 0.227
6	-0.826 / 0.826	1 / 1
7	0.103 / -0.103	1 / 1
8	-3.681 / 3.681	0.009 / 0.009
10	1.396 / -1.396	1 / 1
11	-2.663 / 2.663	0.294 / 0.294
13	9.26 / -9.26	<.001 / <.001
14	-1.346 / 1.346	1 / 1
15	4.506 / -4.506	<.001 / <.001
17	-1.384 / 1.384	1 / 1

Topic	Residuals (country mention / no country mention)	p values (country mention / no country mention)
19	1.123 / -1.123	1 / 1
21	3.128 / -3.128	0.067 / 0.067
22	-0.585 / 0.585	1 / 1
23	-0.267 / 0.267	1 / 1
25	-0.07 / 0.07	1 / 1

Study S4

As mentioned in the discussion of the main manuscript, we only looked for terms referring to country names and demonyms for the main analyses. Terms such as "a cross-cultural study in 30 countries" were not included. To address this specific limitation, we ran an analysis with a series of terms alike (see "S4list_of_terms.csv" file in the repository for a complete list of terms used) on the same dataset. Results are presented below in Table S4.

Results show that there are few mentions of those terms, and they are mostly concentrated in journals related to culture.

Table S4

Percentage of mentions of each region by total articles by journal

Journal	Mentions	Total Articles	Proportion
Annual Review of Psychology	2	638	0.31%
Asian Journal of Social Psychology	4	619	0.65%
British Journal of Social Psychology	3	1,194	0.25%
Culture & Psychology	2	734	0.27%
Current Opinion in Psychology	6	973	0.62%
Deviant Behavior	6	1,301	0.46%
Dynamics of Asymmetric Conflict	1	173	0.58%
Emotion, Space and Society	2	475	0.42%
European Journal of Personality	9	1,048	0.86%
European Journal of Social Psychology	11	2,004	0.55%
Family Science	2	142	1.41%
Group Processes & Intergroup Relations	4	956	0.42%
Intelligence	24	1,517	1.58%

Journal	Mentions	Total Articles	Proportion
International Journal of Intercultural Relations	22	1,364	1.61%
International Journal of Psychology	27	1,497	1.8%
International Journal of Transgenderism	3	289	1.04%
Journal For The Theory of Social Behaviour	1	929	0.11%
Journal of Applied Social Psychology	3	3,622	0.08%
Journal of Bisexuality	3	557	0.54%
Journal of Community & Applied Social Psychology	2	965	0.21%
Journal of Criminal Justice	10	1,584	0.63%
Journal of Cross-Cultural Psychology	79	1,625	4.86%
Journal of Experimental Social Psychology	4	2,750	0.15%
Journal of GLBT Family Studies	4	379	1.06%
Journal of Health and Social Behavior	9	513	1.75%
Journal of Homosexuality	4	2,033	0.2%
Journal of LGBT Youth	3	378	0.79%
Journal of Language and Social Psychology	2	774	0.26%
Journal of Lesbian Studies	1	787	0.13%
Journal of Occupational Science	3	714	0.42%
Journal of Occupational and Social Psychology	4	756	0.53%
Journal of Organizational Behavior	6	1,643	0.37%
Journal of Personality and Social Psychology	23	4,757	0.48%
Journal of Research in Crime and Delinquency	7	630	1.11%
Journal of Research in Personality	6	2,103	0.29%
Journal of Sexual Aggression	8	494	1.62%
Journal of Social Issues	12	1,431	0.84%
Journal of Social and Personal Relationships	2	1,733	0.12%
Personality and Individual Differences	46	10,235	0.45%
Personality and Social Psychology Bulletin	8	3,573	0.22%
Personnel Psychology	5	819	0.61%
Political Psychology	8	1,092	0.73%
PsyCh Journal	3	299	1%

Journal	Mentions	Total Articles	Proportion
Psychological Science	11	4,135	0.27%
Psychological Science in The Public Interest	1	47	2.13%
Psychology and Developing Societies	3	363	0.83%
Psychology and Sexuality	1	294	0.34%
Psychology, Crime & Law	11	1,065	1.03%
Religion, Brain & Behavior	1	150	0.67%
Social Issue and Policy Review	1	107	0.93%
Social Psychological and Personality Science	8	957	0.84%
Social Psychology Quarterly	4	345	1.16%
Social and Personality Psychological Compass	5	900	0.56%
The Journal of Forensic Psychiatry & Psychology	9	892	1.01%

References

Aly, M., Colunga, E., Crockett, M. J., Goldrick, M., Gomez, P., Kung, F. Y. H., McKee, P. C., Pérez, M., Stilwell, S. M., & Diekman, A. B. (2023). Changing the culture of peer review for a more inclusive and equitable psychological science. *Journal of Experimental Psychology: General*, *152*(12), 3546–3565. https://doi.org/10.1037/xge0001461

Amano, T., González-Varo, J. P., & Sutherland, W. J. (2016). Languages Are Still a Major Barrier to Global Science. *PLOS Biology*, *14*(12), e2000933. https://doi.org/10.1371/journal.pbio.2000933

Arnett, J. J. (2008). The Neglected 95%: Why American Psychology Needs to Become Less American. *American Psychologist*, *63*(7), 602–614. https://doi.org/10.1037/0003-066X.63.7.602

Atari, M., Haidt, J., Graham, J., Koleva, S., Stevens, S. T., & Dehghani, M. (2023). Morality beyond the WEIRD: How the nomological network of morality varies across cultures. *Journal of Personality and Social Psychology*, *125*(5), 1157–1188. https://doi.org/10.1037/pspp0000470

Augie, B. (2017). *R package EGG*.

Baumgartner, H. A., Alessandroni, N., Byers-Heinlein, K., Frank, M. C., Hamlin, J. K., Soderstrom, M., Voelkel, J. G., Willer, R., Yuen, F., & Coles, N. A. (2023). How to build up big team science: A practical guide for large-scale collaborations. *Royal Society Open Science*, *10*(6), 230235. https://doi.org/10.1098/rsos.230235

Bettache, K. (2026). “WEIRDly Exceptional”: Has Cultural Psychology Reproduced What It Sought to Critique? *Perspectives on Psychological Science*, 1–15. https://doi.org/10.1177/17456916261474830

Bliese, P. D. (2012). *Mulilevel modeling in R (2.4): A brief introduction to R, the multilevel package and the nlme package*. Walter Reed Army Institute of Research.

Castro Torres, A. F., & Alburez-Gutierrez, D. (2022). North and South: Naming practices and the hidden dimension of global disparities in knowledge production. *Proceedings of the National Academy of Sciences*, *119*(10), e2119373119. https://doi.org/10.1073/pnas.2119373119

Cheon, B. K., Melani, I., & Hong, Y. (2020). How USA-Centric Is Psychology? An Archival Study of Implicit Assumptions of Generalizability of Findings to Human Nature Based on Origins of Study Samples. *Social Psychological and Personality Science*, *11*(7), 928–937. https://doi.org/10.1177/1948550620927269

Dotson, V. M., & Duarte, A. (2020). The importance of diversity in cognitive neuroscience. *Annals of the New York Academy of Sciences*, *1464*(1), 181–191. https://doi.org/10.1111/nyas.14268

Dvorakova, A. (2025). All Psychologies are Indigenous: Addressing Historical Grounding of WEIRD Social Psychological Knowledge in Colonialism and Racism. *Psychological Studies*. https://doi.org/10.1007/s12646-025-00867-w

Egger, R., & Yu, J. (2022). A Topic Modeling Comparison Between LDA, NMF, Top2Vec, and BERTopic to Demystify Twitter Posts. *Frontiers in Sociology*, *7*(May), 1–16. https://doi.org/10.3389/fsoc.2022.886498

Gergen, K. J. (1973). Social psychology as history. *Journal of Personality and Social Psychology*, *26*(2), 309–320. https://doi.org/10.1037/h0034436

Ghai, S., Forscher, P. S., & Chuan-Peng, H. (2024). Big-team science does not guarantee generalizability. *Nature Human Behaviour*, *8*(6), 1053–1056. https://doi.org/10.1038/s41562-024-01902-y

Grootendorst, M. (2022). BERTopic: Neural topic modeling with a class-based TF-IDF procedure. *ArXIv Preprint*, *2203.05794*.

Hadley Wickham. (2016). *ggplot2: Elegant Graphics for Data Analysis*. Springer-Verlag.

Hafeez, D. M., Waqas, A., Majeed, S., Naveed, S., Afzal, K. I., Aftab, Z., Zeshan, M., & Khosa, F. (2019). Gender distribution in psychiatry journals' editorial boards worldwide. *Comprehensive Psychiatry*, *94*, 152119. https://doi.org/10.1016/j.comppsych.2019.152119

Henrich, J., Heine, S. J., & Norenzayan, A. (2010). The weirdest people in the world? *Behavioral and Brain Sciences*, *33*, 61–135. https://doi.org/10.1017/S0140525X10000725

Hommel, B. (2024a). Dealing With Diversity in Psychology: Science or Ideology? *Perspectives on Psychological Science*, *19*(3), 558–563. https://doi.org/10.1177/17456916241236170

Hommel, B. (2024b). Diversity for Truth: Reply to Jussim, Stanovich, and Stroebe. *Perspectives on Psychological Science*, *19*(3), 585–589. https://doi.org/10.1177/17456916241236175

Hornstein, G. A., & Star, S. L. (1990). Universality Biases: How Theories About Human Nature Succeed. *Philosophy of the Social Sciences*, *20*(4), 421–436. https://doi.org/10.1177/004839319002000401

Jakubowski, K., Ahmad, N., Armitage, J., Barrett, L., Edwards, A., Galbo, E., Gómez-Cañón, J. S., Graves, T. A., Jadzgevičiūtė, A., Kirts, C., Lahdelma, I., Lennie, T. M., Ramatally, A., Schlichting, J. L., Steliou, C., Vishwanath, K., & Eerola, T. (2025). Participant and Musical Diversity in Music Psychology Research. *Music & Science*, *8*, 20592043251317180. https://doi.org/10.1177/20592043251317180

Kahalon, R., Klein, V., Ksenofontov, I., Ullrich, J., & Wright, S. C. (2022). Mentioning the Sample's Country in the Article's Title Leads to Bias in Research Evaluation. *Social Psychological and Personality Science*, *13*(2), 352–361. https://doi.org/10.1177/19485506211024036

Krys, K., Cross, S., Adjei, S. B., San Martin, A., Wlodarczyk, A., & Gorski, M. R. (2026). Toward globalizing psychological journals. Actionable recommendations for creating space for research from Majority World contexts. *Psicologia Sociale*, (2), 155–168. https://doi.org/10.1482/121627

Krys, K., Vignoles, V. L., De Almeida, I., & Uchida, Y. (2022). Outside the "Cultural Binary": Understanding Why Latin American Collectivist Societies Foster Independent Selves.

Perspectives on Psychological Science, *17*(4), 1166–1187. https://doi.org/10.1177/17456916211029632

Kuznetsova, A., Brockhoff, P. B., & Christensen, R. H. B. (2022). *Package ' lmerTest '* (3.1-3).

Lui, P. P., Gobrial, S., Pham, S., Giadolor, W., Adams, N., & Rollock, D. (2022). Open science and multicultural research: Some data, considerations, and recommendations. *Cultural Diversity & Ethnic Minority Psychology*, *28*(4), 567–586. https://doi.org/10.1037/cdp0000541

Manalili, M. A. R., Pearson, A., Sulik, J., Creechan, L., Elsherif, M., Murkumbi, I., Azevedo, F., Bonnen, K. L., Kim, J. S., Kording, K., Lee, J. J., Obscura, M., Kapp, S. K., Röer, J. P., & Morstead, T. (2023). From Puzzle to Progress: How Engaging With Neurodiversity Can Improve Cognitive Science. *Cognitive Science*, *47*(2), e13255. https://doi.org/10.1111/cogs.13255

Markus, H. R. (2017). American = Independent? *Perspectives on Psychological Science*, *12*(5), 855–866. https://doi.org/10.1177/1745691617718799

Mook, D. G. (1983). In Defense of External Invalidity. *American Psychologist*, *38*(4), 379–387. https://doi.org/https://doi.org/10.1037/0003-066X.38.4.379

Nair, V. K., Farah, W., & Boveda, M. (2026). Is neurodiversity a Global Northern White paradigm? *Autism*, *30*(2), 544–551. https://doi.org/10.1177/13623613241280835

Nielsen, M., Haun, D., Kärtner, J., & Legare, C. H. (2017). The persistent sampling bias in developmental psychology: A call to action. *Journal of Experimental Child Psychology*, *162*, 31–38. https://doi.org/10.1016/j.jecp.2017.04.017

Palser, E. R., Lazerwitz, M., & Fotopoulou, A. (2022). Gender and geographical disparity in editorial boards of journals in psychology and neuroscience. *Nature Neuroscience*, *25*(3), 272–279. https://doi.org/10.1038/s41593-022-01012-w

Pepitone, A., & Triandis, H. C. (1987). On the Universality of Social Psychological Theories. *Journal of Cross-Cultural Psychology*, *18*(4), 471–498. https://doi.org/10.1177/0022002187018004003

Pitesa, M., & Gelfand, M. J. (2023). Going beyond Western, Educated, Industrialized, Rich, and Democratic (WEIRD) samples and problems in organizational research. *Organizational Behavior and Human Decision Processes*, *174*, 104212. https://doi.org/10.1016/j.obhdp.2022.104212

Pollet, T. V., & Saxton, T. K. (2019). How Diverse Are the Samples Used in the Journals 'Evolution & Human Behavior' and 'Evolutionary Psychology'? *Evolutionary Psychological Science*, *5*(3), 357–368. https://doi.org/10.1007/s40806-019-00192-2

Puthillam, A., Montilla Doble, L. J., Delos Santos, J. J. I., Elsherif, M. M., Steltenpohl, C. N., Moreau, D., Pownall, M., Silverstein, P., Anand-Vembar, S., & Kapoor, H. (2024). Guidelines to improve internationalization in the psychological sciences. *Social and Personality Psychology Compass*, *18*(1), e12847. https://doi.org/10.1111/spc3.12847

R Development Core Team. (2023). *R: A Language and Environment for Statistical Computing* (4.2.3). R Foundation for Statistical Computing.

Rad, M. S., Martingano, A. J., & Ginges, J. (2018). Toward a psychology of *Homo sapiens*: Making psychological science more representative of the human population. *Proceedings of the National Academy of Sciences*, *115*(45), 11401–11405. https://doi.org/10.1073/pnas.1721165115

Richmond, A. S., Broussard, K. A., Sterns, J. L., Sanders, K. K., & Shardy, J. C. (2015). Who Are We Studying? Sample Diversity in Teaching of Psychology Research. *Teaching of Psychology*, *42*(3), 218–226. https://doi.org/10.1177/0098628315587619

Rozin, P. (2001). Social Psychology and Science: Some Lessons From Solomon Asch. *Personality and Social Psychology Review*, *5*(1), 2–14. https://doi.org/10.1207/S15327957PSPR0501_1

Said, E. W. (1978). *Orientalism* (1st ed). Pantheon Books.

Schimmelpfennig, R., Elbæk, C., Mitkidis, P., Singh, A., & Roberson, Q. (2025). The “WEIRDEST” Organizations in the World? Assessing the Lack of Sample Diversity in Organizational Research. *Journal of Management*, *51*(6), 2460–2487. https://doi.org/10.1177/01492063241305577

Sherman, J. W. (2025). There is nothing WEIRD about basic research: The critical role of convenience samples in psychological science. *American Psychologist*, *80*(7), 1073–1080. https://doi.org/10.1037/amp0001443

Solomonov, N., Chen, S. Z., Briskin, E. A., Castonguay, L., Krause, M., McMain, S., Duggal, C., Youn, S. J., Lorenzo-Luaces, L., & Barber, J. P. (2025). A first look at diversity gaps in psychotherapy research publications and representation. *Psychotherapy Research*, *35*(8), 1275–1286. https://doi.org/10.1080/10503307.2024.2428693

Stroebe, W. (2024). The Myth of the Need for Diversity Among Subjects in Theory-Testing Research: Comments on “Racial Inequality in Psychological Research” by Roberts et al. (2020). *Perspectives on Psychological Science*, *19*(3), 576–579. https://doi.org/10.1177/17456916241236165

Thalmayer, A. G., Toscanelli, C., & Arnett, J. J. (2020). The Neglected 95% Revisited: Is American Psychology Becoming Less American? *American Psychologist*, 1–36. https://doi.org/10.1037/amp0000622

Tindle, R. (2021). Improving the global reach of psychological research. *Discover Psychology*, *1*(1), 5. https://doi.org/10.1007/s44202-021-00004-4

Uskul, A. K., & Mesquita, B. (2026). Advancing a dynamic approach to cultural psychology beyond stable dichotomies. *Nature Reviews Psychology*. https://doi.org/10.1038/s44159-026-00550-5

Vaidis, D. C., Miranda, J. F., Buchanan, E. M., Yang, Y.-F., Kowal, M., Schmidt, K., Topor, M., Misiak, M., Miller, R., Protzko, J., Gjoneska, B., Miller, J. K., Exner, A., Azevedo, F., Paruzel-Czachura, M., Mushtaq, F., Oliveira, C. M., Wagge, J. R., De Moor, D., … Pronizius, E. (2024). *The Advantage of Big Team Science: Lessons Learned from Cognitive Science*. Open Science Framework. https://doi.org/10.31219/osf.io/yvm5h

Van Rossum, G., & Drake, F. L. (2011). *Python language reference manual*. Network Theory Ltd.

Vignoles, V. L. (2018). The “common view”, the “cultural binary”, and how to move forward. *Asian Journal of Social Psychology*, *21*(4), 336–345. https://doi.org/10.1111/ajsp.12346

Watts, R. J. (1992). Elements of a psychology of human diversity. *Journal of Community Psychology*, *20*(2), 116–131. https://doi.org/10.1002/1520-6629(199204)20:2%3C116::AID-JCOP2290200203%3E3.0.CO;2-S

Webster, G. D., Mahar, E. A., & Wongsomboon, V. (2021). American psychology is becoming more international, but too slowly: Comment on Thalmayer et al. (2020). *American Psychologist*, *76*(5), 802–805. https://doi.org/10.1037/amp0000747

Webster, G. D., Nichols, A. L., & Schember, T. O. (2009). American psychology is becoming more international. *American Psychologist*, *64*(6), 566–568. https://doi.org/10.1037/a0016193
